# Epidemiological features and risk factors associated with the spatial and temporal distribution of human brucellosis in China

**DOI:** 10.1186/1471-2334-13-547

**Published:** 2013-11-16

**Authors:** Yin-Jun Li, Xin-Lou Li, Song Liang, Li-Qun Fang, Wu-Chun Cao

**Affiliations:** 1 State Key Laboratory of Pathogens and Biosecurity, Beijing Institute of Microbiology and Epidemiology, 20 Dong-Da Street, Fengtai District, Beijing 100071, People’s Republic of China; 2 Department of Environmental and Global Health, College of Public Health and Health Professions, and Emerging Pathogens Institute, University of Florida, Gainesville, Florida, USA

**Keywords:** Infectious diseases, Pandemics, Disease outbreaks, Risk assessment, Public health

## Abstract

**Background:**

Human brucellosis incidence in China has been increasing dramatically since 1999. However, epidemiological features and potential factors underlying the re-emergence of the disease remain less understood.

**Methods:**

Data on human and animal brucellosis cases at the county scale were collected for the year 2004 to 2010. Also collected were environmental and socioeconomic variables. Epidemiological features including spatial and temporal patterns of the disease were characterized, and the potential factors related to the spatial heterogeneity and the temporal trend of were analysed using Poisson regression analysis, Granger causality analysis, and autoregressive distributed lag (ADL) models, respectively.

**Results:**

The epidemic showed a significantly higher spatial correlation with the number of sheep and goats than swine and cattle. The disease was most prevalent in grassland areas with elevation between 800–1,600 meters. The ADL models revealed that local epidemics were correlated with comparatively lower temperatures and less sunshine in winter and spring, with a 1–7 month lag before the epidemic peak in May.

**Conclusions:**

Our findings indicate that human brucellosis tended to occur most commonly in grasslands at moderate elevation where sheep and goats were the predominant livestock, and in years with cooler winter and spring or less sunshine.

## Background

Brucellosis is a zoonosis caused by bacteria of the *Brucella spp.* Infections in humans can cause an allergic reaction, characterised by a 1–2 month incubation period followed by prolonged fever, night sweats, body aches, arthralgia, and weakness [1,2]. Human infections are typically through consumption of raw milk or unpasteurized cheese contaminated by the bacterial agent, and to a less extent, contact with infected animals [3-5]. Person*-*to*-*person transmission of *Brucella* is extremely rare [6]. Farmers, shepherds, abattoir and veterinary workers have traditionally been considered to be high risk occupations [7]. Sheep, goats, cattle, swine, and dogs, are all susceptible to infection, serving as natural hosts. *B. melitensis*, *B. abortus, B. suis* and *B. canis* often cause abortion and infertility in these natural hosts [4,8-11]. *B. melitensis* Rev.1 is an effective commercial vaccine which is currently used for small ruminants, and no satisfactory vaccine against human infection is available [12,13]. Brucellosis has a worldwide distribution in both humans and animals with the exception of several industrialized countries, causing tremendous health and economic burden [14]. Worldwide, governmental and health agencies of affected countries have made extensive efforts e to control brucellosis primarily through the culling of infected animals and regulations for safe disposal of infected material [15,16]. Nevertheless, human brucellosis has taken a heavy toll on the health and economy of countries affected and it must still be regarded as a serious worldwide public health problem.

Human brucellosis remains one of major public health issues in China. At present, human brucellosis is endemic in 25 of 32 provinces or autonomous regions of China [9]. Human brucellosis cases have been reported since the mid-1950s in China, the Inner Mongolia Autonomous Region being the most severely affected since 1999 [17]. Since the beginning of the 21st century, human brucellosis incidence has risen dramatically. The number of reported cases increased each year until 2009 and was among the top 10 notifiable infectious diseases during 2000–2006 in terms of the total number of cases reported [18].

Human Brucellosis infection has previously been associated with animal habitat, occupation, host density, socioeconomic status, travel and immigration [19-23]. China has a great variety of ecological, environmental and economic landscapes across the country. Given the alarming increases in the human cases, there is an urgent need to understand what may underlie the emergence. In this study, we aim to characterise the epidemiological features of human brucellosis and identify environmental and socioeconomic factors associated with spatial patterns of the disease, and to explore the meteorological factors associated with temporal trends in human brucellosis incidence in mainland China from 2004 to 2010.

## Methods

### Data collection and management

The 7-year data set of all human brucellosis cases from 2004 to 2010 in mainland China was obtained from the National Notifiable Disease Surveillance System (NNDSS). The information included age, gender, occupation and month of onset for each patient. In China, human brucellosis is a class B notifiable infectious disease, and information regarding each suspected or confirmed case must be reported to the Chinese CDC (CCDC) through the NNDSS [24]. To satisfy case definitions, a confirmed case must be accompanied by clinical signs including fever lasting several days or weeks, sweating, fatigue and muscle and joint pain, and should also be confirmed by serological tests using the standard plate agglutination test (PAT) and/or rose bengal plate test (RBPT) and/or serum agglutination test (SAT), or bacterial isolation in accordance with the case definition of the World Health Organization. The data on animal husbandry, environment, and factors including livestock density (sheep, goats, swine and cattle), average elevation, vegetation coverage (croplands, forests, and grassland), and climate variables were collected. Average elevation and livestock density data for China at a nominal resolution of 1 × 1 km and 5 × 5 km were obtained for 2009 and 2005, respectively [25,26]. To extract the area percentages occupied by croplands, forests and grassland in each county, the land cover data from 2005 were collected [27]. The average monthly meteorological data including temperature, rainfall, hours of sunshine (HS), relative humidity (RH) and average wind velocity (WV), were extracted from 130 national meteorological monitoring stations for the four provinces with highest human brucellosis epidemics Inner Mongolia, Heilongjiang, Shanxi and Jilin) [28]. The present study was reviewed and approved by the research institutional review board (IRB) of Beijing Institute of Microbiology and Epidemiology. Due to absence of personal identifiers in the surveillance data and aggregate nature of the data, the IRB waived requirement of informed consent. Readers interested in further research can contact the corresponding author to obtain the full dataset used in this study.

### Analyses of epidemiological features

To characterise the epidemiological features of human brucellosis in mainland China, a monthly incidence histogram with annual incidence curve was produced, as well as annual incidence histograms for gender and age groups using data from 2004 to 2010. The proportion of human cases according to occupation was mapped using data from 2010. To assess spatiotemporal distribution and trends of human brucellosis, the annual incidence of each county was calculated and mapped. In addition, a map of animal brucellosis cases in 2004–2010 was created.

### Analysis of factors associated with spatial pattern of human brucellosis incidence

The environmental and socioeconomic factors including the number of livestock (sheep, goats, swine, and cattle), average elevation and the area percentage occupied by croplands, forests and grassland, associated with the spatial pattern of human brucellosis incidence, for the 2,922 counties were calculated by using ArcMap version 9.3. We applied a Poisson regression framework to explore the associations between the incidence of human brucellosis and environmental and socioeconomic factors at county level. The cumulative number of human brucellosis cases for each county from 2004 to 2010 was set as the outcome variable, and the number of population was included as the offset. Potential environmental and socioeconomic factors, such as numbers of sheep, goats, swine and cattle, average elevation, and the area proportions of croplands, forests and grassland for each county were included as co-variables in the analysis. The incidence rate ratio (IRR) in response to the change of a variable by a given amount (100,000 head for sheep, goats, swine and cattle, 10% for the area proportion of croplands, forests and grassland, and categorical variable for average elevation), was used to determine the impact of each variable on disease incidence. The 95% confidence interval (CI) and corresponding P-value were estimated after correcting for the over-dispersion. In this study, the variance of cumulative number of human brucellosis cases was larger than the mean and the over-dispersion parameter “α” was tested to be significantly different from zero, which indicated the need to correct for over-dispersion, and the square root of the Pearson chi-square dispersion was used to scale the standard errors [29]. Univariate analyses were performed to examine the effect of each variable separately, and then multivariate analysis was performed by including all co-variables with a *p* < 0.20 in the univariate analysis. Correlations between co-variables were quantitatively assessed and models would be optimized if high collinearity (spearman correlation coefficients > 0.7) was found. The analysis was performed in STATA 9.1 software (StataCorp LP, College Station TX, USA).

### Analysis of meteorological factors associated with the temporal trend of incidence of human brucellosis

To explore the probable factors influencing the re-emergence of human brucellosis in mainland China, cross-correlation analyses were conducted to examine the association between the monthly incidence of human brucellosis and each of the climatic variables for the four provinces which had the top four cumulative incidence from 2004 to 2010. Lag times (in months) for climatic variables were used in the analysis to explore any lagged effects. The lag time with maximum correlation coefficient between human brucellosis and climatic variables as well as the lag times with significant correlation between them were identified. To further analyse the probable effect of climatic factors on human brucellosis incidence, the Granger causality tests for climate variables influencing monthly incidence of human brucellosis were performed based on the lag times with significant correlation between them in the cross-correlation analyses. The variables of Granger causality for human brucellosis transmission were identified and then the autoregressive distributed lag (ADL) models based on these variables were carried out to examine the contribution of climatic factors to human brucellosis transmission for the four provinces. The ADL model was constructed as follows:

$$\begin{array}{l} Y_{t}=a+b_{0}X_{t}+b_{1}X_{t-1}+b_{2}X_{t-2}+\ldots +b_{k}X_{t-k}\\ \;+b_{q}Y_{t-1}+u_{t} \end{array}$$

where the coefficients *b*_*i*_ (i = 0,1,2…*k*) describe the lagged effects of *X* on *Y, b*_*q*_is the autoregressive coefficient of Y(t-1), and *u*_*t*_ which represents the residual. Letting *b*_*i*_ represent a polynomial of degree *m* in *i*:

$$b_{i}=\alpha_{0}+\alpha_{1}i+\alpha_{2}i^{2}+\alpha_{3}i^{3}+.....+\alpha_{m}i^{m}\;\left(m<k\right)$$

then,

$$\begin{array}{l} Y_{t}=a+a_{0}z_{0t}+a_{1}z_{1t}+a_{2}z_{2t}+\ldots +a_{m}z_{\mathrm{mt}}+b_{q}Y_{t-1}\\ \;+u_{t},\mathrm{and}\;z_{\mathrm{jt}}\\ \;=\sum_{i=0}^{k}i^{j}x_{t-i},j=0,1,\ldots ,m. \end{array}$$

In this study, the monthly incidence of human brucellosis was used as a dependent variable, and monthly meteorological variables (temperature, rainfall, HS, RH, and WV) were used as the independent variables. Akaike’s information criterion (AIC) was used to identify the lags of meteorological variables and measure goodness-of-fit of the ADL models. The predictive validity of the models was evaluated using the root mean square error ($\mathrm{RMSE}=\sqrt{\sum_{t=1}^{N}{\left({\hat{Y}}_{t}-Y_{t}\right)}^{2}/N}$, where Ŷ_t_ is the predicted value for month t, Y_t_ is the observed value, and N is the number of observations). Also, we performed the first order autoregressive model to provide useful information for understanding the contribution resulting from climatic factors. The data spanning the period January 2004 to December 2009 were used to construct and optimise the models, while the data from January 2010 to December 2010 were used to assess the predictive ability of the models.

## Results

### Epidemiological features of human brucellosis in mainland China

**Figure 1:**
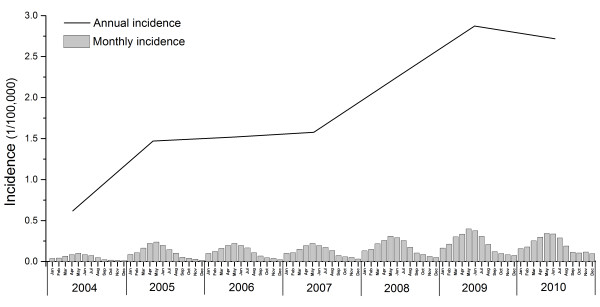
Temporal distribution of human brucellosis in the mainland China, 2004–2010.

**Figure 2:**
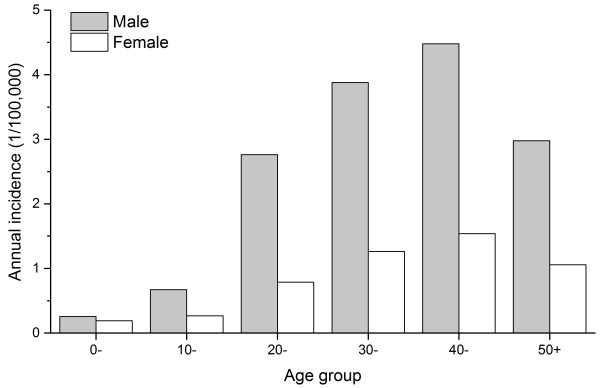
Human brucellosis incidence over gender and age group in mainland China.

**Figure 3:**
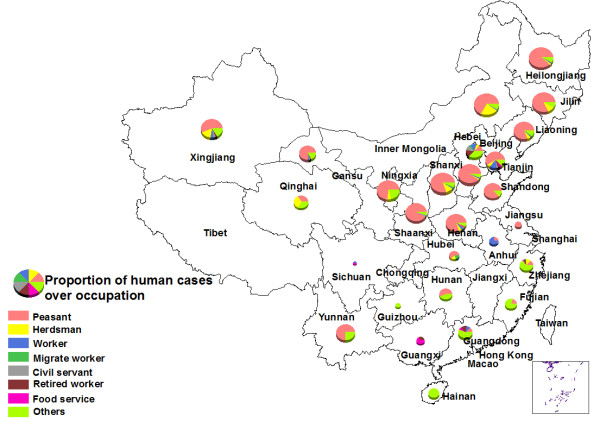
Distribution map of proportion of human cases over occupation (2010).

A total of 162,329 cases were reported from 2004 to 2010, distributed across 1,201 of 2,922 counties (41%). The annual incidence had sharply increased by approximately 4 times from 0.63 to 2.72 per 100,000 person years during the 7-year period, and the monthly incidence showed a significant seasonal pattern peaking in the spring and summer season, especially in the month of May each year (Figure 1). 74.59% of all reported cases occurred in males, and males had a significantly higher incidence than females in all age groups (p value < 0.001). The bulk of the cases (51-54%) occurred in the 30–49 age group (Figure 2). In addition, 88.78% of all cases came from peasant and herdsman, and patients’ occupations showed differences between northern and southern China. Mostly patients came from peasant and herdsman in northern, north-eastern and north-western China, especially in the pastoral and agricultural regions, while patients from other occupations such as food services, city workers and retired workers predominated in southern China or in more urban areas (Figure 3).

**Figure 4:**
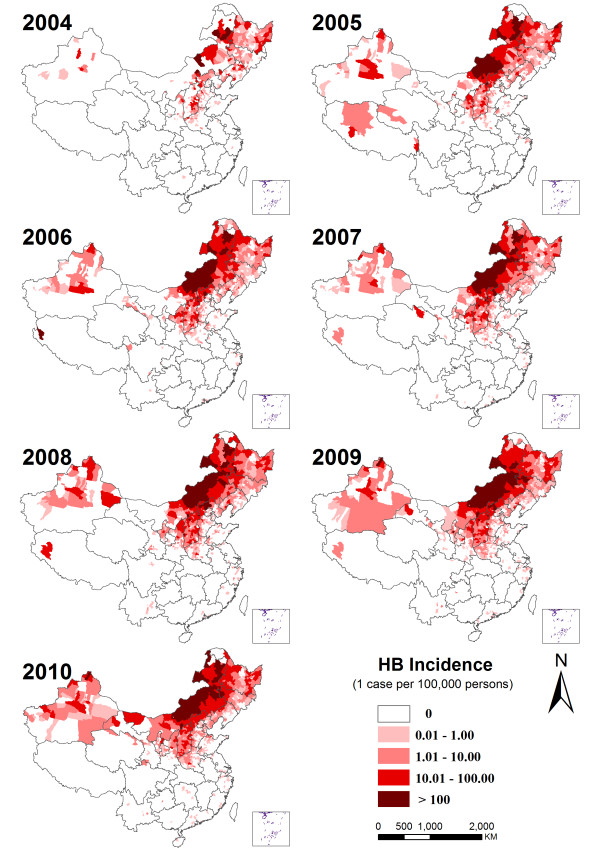
Spatiotemporal distribution of human brucellosis incidence in mainland China, 2004–2010.

The spatiotemporal distribution map showed that human brucellosis was widely distributed in the provinces of Inner Mongolia, Hebei and Shanxi in northern China, and Heilongjiang, Jilin and Liaoning provinces in north-eastern China. Xinjiang Autonomous Region in north-western China also showed an increase in incidence over the 7 years. Other provinces, especially in eastern and southern China, showed a more sporadic occurrence of the disease (Figure 4). At county level, the annual incidence ranged from 0 to1440 cases per 100,000 person years with a mean of 11. The three counties with highest average annual incidence were Sonidzuo Qi, Abag Qi and Xianghuang Qi in Inner Mongolia (1,440, 1,121 and 902 per 100,000 person years, respectively). The spatiotemporal map also showed that the extent of epidemic areas of human brucellosis had expanded since 2005, especially in the western and northern areas of China, to reach the historic high in 2009. Meanwhile, the extent of high incidence in northern China had also tended to move southward.

### Factors associated with spatial pattern of human brucellosis incidence

Univariate Poisson regression analyses revealed that human brucellosis incidence was significantly associated with the number of sheep, goats and swine, in grassland areas of average elevation. It was not significantly associated with the number of cattle, and the percentage area occupied by croplands and forests. Multivariate analysis including all co-variables with a *p* < 0.20 in univariate analysis, demonstrated that five variables - number of sheep, number of goats, number of swine, average elevation and the percentage area occupied by grassland, were significantly associated with human brucellosis outbreaks (Table 1). The table shows the adjusted figures between human brucellosis incidence and livestock. The IRR for the number of sheep, goats and swine were 1.42 (95% CI = 1.28 – 1.57, *p* <0.001), 1.15 (95% CI = 1.01 -1.32, *p* = 0.033) and 0.80 (95% CI = 0.66 – 0.96, *p* = 0.016) respectively. Brucellosis incidence was also influenced by elevation and vegetation, and the counties with moderate elevation (800–1,600 meters) and more grassland showed a higher IRR.

**Table 1:** The association between brucellosis incidence and influencing factors by Poisson regression

**Variables (unit) **^**(a)**^	**Average yearly incidence (95% CI, per 100,000 person-years)**	**Univariate analysis**		**Multivariate analysis**	
		**Crude IRR (95% CI)**	**P-value**	**Adjusted IRR**^**(b) **^**(95% CI)**	**P-value**
**Sheep** (categorical, 1000 heads)					
< 0.03	0.23 (0.03 - 0.43)				
0.03 -	1.00 (0.63 - 1.36)				
> 8	14.30 (9.76 - 18.84)				
**Sheep** (continuous, 100,000 heads)		1.54 (1.48 - 1.60)	< 0.001	1.42 (1.28 - 1.57)	< 0.001
**Goats** (categorical, 1000 heads)					
< 0.3	0.95 (0.63 - 1.27)				
0.3 -	3.56 (2.38 - 4.74)				
> 3	10.10 (5.87 - 14.33)				
**Goats** (continuous, 100,000 heads)		1.41 (1.22 - 1.62)	< 0.001	1.15 (1.01 - 1.32)	0.033
**Swine** (categorical, 1000 heads)					
< 30	7.79 (3.91 - 11.67)				
30 -	4.28 (2.96 - 5.59)				
> 150	1.95 (1.11 - 2.78)				
**Swine** (continuous, 100,000 heads)		0.64 (0.48 - 0.87)	0.004	0.80 (0.66 - 0.96)	0.016
**Cattle** (categorical, 1000 heads)					
< 3	1.23 (0.77 - 1.70)				
3 -	4.82 (2.86 - 6.78)				
> 30	8.49 (4.43 - 12.56)				
**Cattle** (continuous, 100,000 heads)		1.44 (0.95 - 2.19)	0.084	0.70 (0.44 - 1.11)	0.126
**Elevation** (categorical, 1000 m)					
< 0.4	1.59 (0.90 - 2.28)	1	-	1	-
0.4 -	4.17 (2.17 - 6.18)	2.97 (1.44 - 6.16)	0.003	3.31 (1.93 - 5.68)	< 0.001
0.8 -	17.47 (10.25 - 24.68)	10.12 (5.78 - 17.72)	< 0.001	5.77 (3.68 - 9.05)	< 0.001
> 1.6	0.78 (0.07 - 1.49)	0.70 (0.12 - 4.23)	0.701	0.44 (0.12 - 1.69)	0.234
**Croplands** (categorical,%)^§^					
< 20	7.26 (3.29 - 11.23)				
20 -	3.97 (2.97 - 4.96 )				
> 50	3.05 (1.85 - 4.25 )				
**Croplands** (continuous, 10%)		1.00 (0.99 - 1.00)	0.758		
**Forests** (categorical,%)^§^					
< 5	7.75 (3.70 - 11.81)				
5 -	5.46 (3.98 - 6.93)				
> 40	1.25 (0.75 - 1.76 )				
**Forests** (continuous, 10%)		1.00 (0.99 - 1.00)	0.976		
**Grassland** (categorical,%)^§^					
< 2	0.28 (0.20 - 0.37 )				
2 -	2.09 (1.50 - 2.68 )				
> 20	13.33 (8.66 - 18.01 )				
**Grassland** (continuous, 10%)		1.03 (1.01 - 1.04)	< 0.001	1.03 (1.01 - 1.04)	< 0.001

^(a)^ For all continuous variables, we also report categorical results to allow inspection of the data and whether or not the assumption of continuous variables was justified.

^(b)^ Adjusted IRR was corrected for over-dispersion in multivariate analysis. ^§^ The area percentage occupied by croplands, forests or grassland, respectively.

### Meteorological factors associated with the temporal trend of incidence of human brucellosis

Cross-correlation analyses showed that the monthly incidence of human brucellosis was significantly correlated with climatic variables, including temperature, rainfall, HS, RH and WV in all provinces. In the four provinces with highest incidence, a cumulative effect was observed with lag times ranging from 0 to 7 months (Table 2). With the exception of WV, the climatic variables showed negative correlation with disease incidence. Monthly average temperatures with a 4-month lag time were found to have the highest correlation with the incidence of human brucellosis, followed by Rainfall, HS, RH, and WV. Longer lag times were found for temperature, rainfall and HS (3–4 months lags), than for RH and WV (1–2 months lags). Temperature, HS and rainfall showed the highest probabilities of influencing brucellosis transmission in the four provinces according to the Granger causality tests (Table 3).

**Table 2:** Correlation coefficient between the monthly incidence of human brucellosis and climate variables, 2004–2010

**Province**	**Temperature**	**Rainfall**	**HS**	**RH**	**WV**
Inner Mongolia	L4 = −0.70	L4 = −0.58	L4 = −0.69	L1 = −0.50	L1 = 0.51
Heilongjiang	L4 = −0.76	L4 = −0.59	L4 = −0.53	L1 = −0.34	L1 = 0.28
Shanxi	L4 = −0.71	L3 = −0.63	L5 = −0.56	L2 = −0.57	L2 = 0.53
Jilin	L4 = −0.48	L4 = −0.39	L4 = −0.29	L3 = −0.36	L1 = 0.30

The maximum correlation coefficient were shown in the table.

Lx: the lagged months. HS: monthly hours of sunshine; RH: average relative humidity; WV: average wind velocity.

**Table 3:** Granger causality tests for climate variables causing the monthly incidence of human brucellosis.

**Province**	**Temperature**	**Rainfall**	**HS**	**RH**	**WV**
Inner Mongolia	6.28 (< 0.001)	1.95 (0.076)	6.57 (< 0.001)	1.80 (0.172)	3.78 (0.008)
Heilongjiang	6.21 (< 0.001)	2.87 (0.021)	3.01 (0.012)	1.19 (0.310)	0.97 (0.412)
Shanxi	6.41 (< 0.001)	3.60 (0.004)	3.58 (0.003)	1.79 (0.141)	3.97 (0.006)
Jilin	3.30 (0.010)	3.28 (0.010)	0.56 (0.782)	1.98 (0.107)	0.18 (0.833)

The F-statistics (P-value) were shown in the table.

HS: monthly hours of sunshine; RH: average relative humidity; WV: average wind velocity.

**Figure 5:**
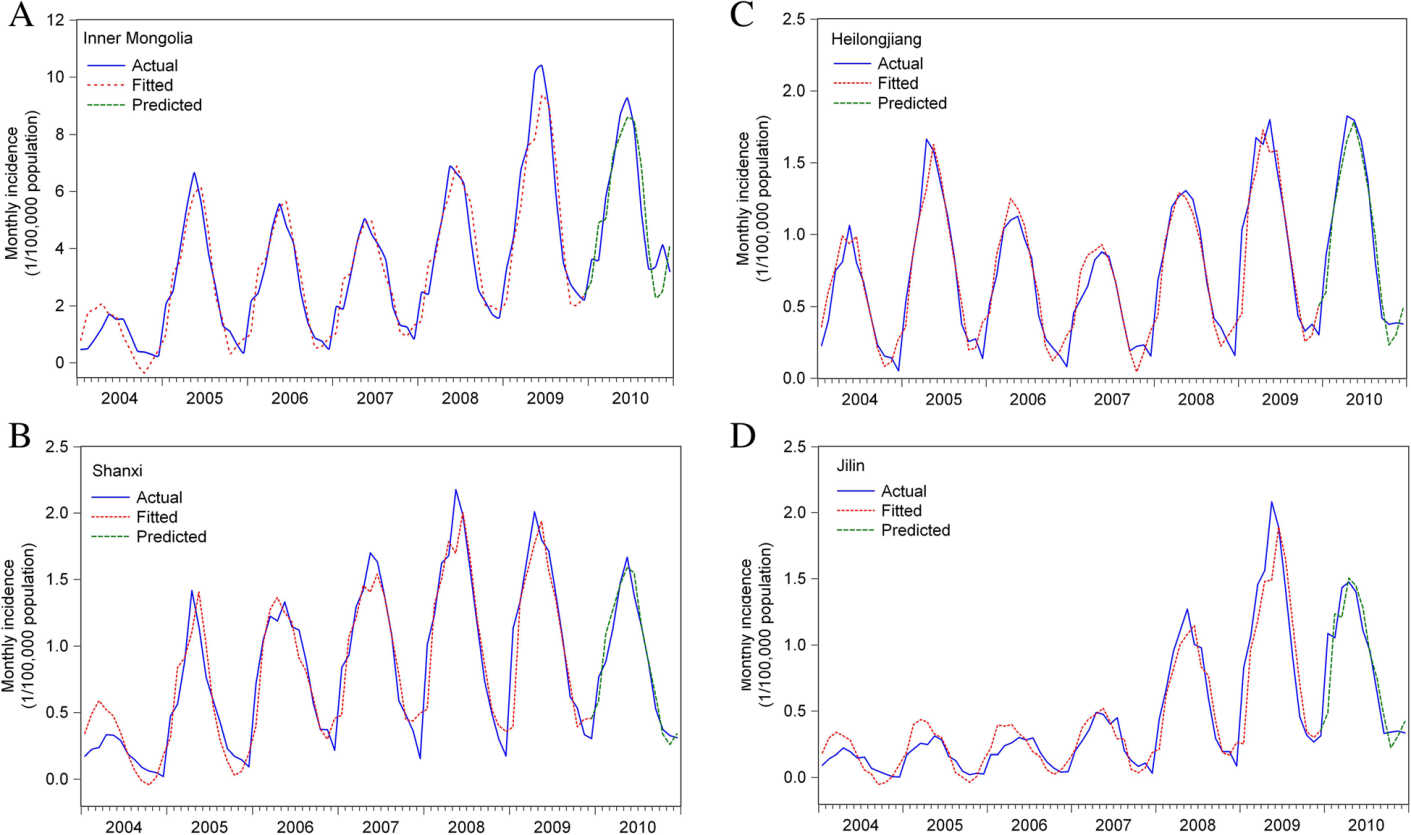
**Validations of ADL models of human brucellosis incidence in provinces with the highest incidences, China. A.** Inner Mongolia Autonomous Region, northern China; B. Shanxi Province, northern China**; C.** Heilongjiang Province, northeastern China; **D.** Jilin Province, northeastern China.

The ADL time-series regression analyses revealed that the incidence of the disease was significantly associated with temperature or HS, or both, with lags of 1–7 months in all four provinces (Table 4). The two variables, rainfall and WV, were excluded from the final (or multivariate) ADL models, because of their minimal contribution to monthly incidence. This model yielded the best fit according to the root mean square error (RMSE). In the inner Mongolian region, the model (Model II, including only HS in the model), incidence was significantly associated with HS, with lag times from 0 to 5 months (β from −0.03 to 0.11 per 10 hours change, approximately 1 day) (Table 4). In Heilongjiang and Jilin provinces, Model I, which included temperature in the model, yielded the best fit and showed that monthly incidence of human brucellosis was significantly associated with temperature at lags from 0 to 6 and from 0 to 7 months, respectively (β from −0.05 to - 0.002 in Heilongjiang per 1°C change, and β from −0.004 to −0.001 in Jilin). Model III including both factors with lags from 0 to 7 months in the model showed the best fit in Shanxi province (β from −0.003 to −0.007 for temperature with lags from 0 to 5 months per 1°C change, and β from −0.007 to −0.006 for HS with lags from 0 to 7 months per 10 hours change). The validation of these ADL models using data from January to December of 2010 demonstrated a good fit between observations and predictions, and the high predictive powers of these models were achieved using the 12-month observations in all four provinces (Figure 5).

**Table 4:** ADL time-series regression coefficients of the temperature and HS associated with human brucellosis, 2004–2009

	**Variables**^**a**^	**Model I**	**Model II**	**Model III**	
		**β(95% CI) for temperature**	**β(95% CI) for HS**	**β(95% CI) for temperature**	**β(95% CI) for HS**
Inner	Lag0	−0.019 (−0.023 to −0.016)	−0.028 (−0.043 to −0.013)	−0.019 (−0.031 to −0.008)	0.033 (−0.011 to 0.076)
Mongolia^b^	Lag1	−0.019 (−0.021 to −0.017)	−0.044 (−0.055 to −0.033)	−0.015 (−0.024 to −0.006)	0.009 (−0.024 to 0.043)
	Lag2	−0.018 (−0.020 to −0.017)	−0.060 (−0.067 to −0.052)	−0.011 (−0.018 to −0.004)	−0.014 (−0.041 to 0.013)
	Lag3	−0.018 (−0.020 to −0.016)	−0.076 (−0.082 to −0.069)	−0.007 (−0.013 to −0.0003)	−0.037 (−0.063 to −0.011)
	Lag4	−0.017(−0.020 to −0.015)	−0.091 (−0.100 to −0.082)	−0.002 (−0.005 to −0.010)	−0.060 (−0.091 to −0.029)
	Lag5	−0.017(−0.021 to −0.013)	−0.107 (−0.120 to −0.094)	0.002 (−0.008 to 0.012)	−0.083 (−0.123 to −0.043)
	Constant term	1.152 (0.946 to 1.357)	10.738 (9.778 to 11.698)	4.706 (1.037 to 8.374)	
	Incidence(−1)	0.846 (0.795 to 0.898)	0.822 (0.772 to 0.873)	0.832 (0.784 to 0.880)	
	R-square	0.925	0.926	0.923	
	AIC	2.088	2.066	2.219	
	RMS error	1.300	1.230	1.180	
Heilongjiang^c^	Lag0	−0.005 (−0.006 to −0.005)	−0.005 (−0.008 to −0.001)	−0.005 (−0.007 to −0.004)	0.005 (−0.0003 to 0.010)
	Lag1	−0.005 (−0.005 to −0.004)	−0.009 (−0.012 to −0.007)	−0.005 (−0.006 to −0.004)	0.003 (−0.002 to 0.007)
	Lag2	−0.004 (−0.005 to −0.004)	−0.014 (−0.016 to −0.012)	−0.004 (−0.005 to −0.003)	0.001 (−0.004 to 0.005)
	Lag3	−0.004 (−0.004 to −0.003)	−0.019 (−0.021 to −0.017)	−0.003 (−0.004 to −0.002)	−0.002 (−0.006 to 0.003)
	Lag4	−0.003 (−0.004 to −0.002)	−0.024 (−0.027 to −0.020)	−0.002 (−0.003 to −0.002)	−0.004 (−0.009 to 0.001)
	Lag5	−0.002 (−0.003 to −0.001)	-	−0.002 (−0.003 to −0.001)	-
	Lag6	−0.002 (−0.003 to −0.001)	-	−0.001 (−0.002 to −0.001)	-
	Constant term	0.267 (0.196 to 0.339)	1.669 (1.494 to 1.844)	0.206 (−0.239 to 0.650)	
	Incidence(−1)	0.741 (0.659 to 0.823)	0.776 (0.716 to 0.835)	0.745 (0.659 to 0.830)	
	R-square	0.910	0.880	0.913	
	AIC	−0.969	−0.708	−0.938	
	RMS error	0.213	0.258	0.216	
**Province**	**Variables**^**a**^	**Model I**	**Model II**	**Model III**	
		**β(95% CI) for temperature**	**β(95% CI) for HS**	**β(95% CI) for temperature**	**β(95% CI) for HS**
Shanxi^d^	Lag0	−0.007 (−0.008 to −0.005)	−0.006 (−0.010 to −0.002)	−0.003 (−0.005 to −0.0003)	−0.007 (−0.013 to −0.0004)
	Lag1	−0.007 (−0.007 to −0.006)	−0.001 (−0.013 to −0.006)	−0.004 (−0.005 to −0.002)	−0.007 (−0.012 to −0.002)
	Lag2	−0.006 (−0.007 to −0.005)	−0.013 (−0.015 to −0.010)	−0.005 (−0.005 to −0.004)	−0.007 (−0.011 to −0.003)
	Lag3	−0.006 (−0.006 to −0.005)	−0.016 (−0.019 to −0.014)	−0.005 (−0.006 to −0.004)	−0.007 (−0.010 to −0.003)
	Lag4	−0.005 (−0.006 to −0.004)	−0.020 (−0.022 to −0.017)	−0.006 (−0.008 to −0.004)	−0.007 (−0.010 to −0.003)
	Lag5	−0.005 (−0.006 to −0.004)	−0.023 (−0.026 to −0.020)	−0.007 (−0.010 to −0.004)	−0.007 (−0.010 to −0.003)
	Lag6	-	−0.026 (−0.031 to −0.022)	-	−0.006 (−0.011 to −0.002)
	Lag7	-	−0.030 (−0.035 to −0.025)	-	−0.006 (−0.012 to −0.001)
	Constant term	0.554 (0.468 to 0.640)	3.230 (2.818 to 3.642)	1.676 (1.16 to 2.19)	
	Incidence(−1)	0.781 (0.716 to 0.846)	0.540 (0.457 to 0.622)	0.609 (0.519 to 0.698)	
	R-square	0.893	0.860	0.903	
	AIC	−0.376	−0.104	−0.506	
	RMS error	0.253	0.303	0.213	
Jilin^e^	Lag0	−0.004 (−0.004 to −0.003)			
	Lag1	−0.003 (−0.004 to −0.003)			
	Lag2	−0.003 (−0.003 to −0.002)			
	Lag3	−0.002 (−0.002 to −0.002)			
	Lag4	−0.001 (−0.002 to −0.001)			
	Lag5	−0.001 (−0.002 to −0.0001)			
	Constant term	0.123 (0.090 to 0.157)			
	Incidence(−1)	0.914 (0.867 to 0.962)			
	R-square	0.894			
	AIC	−0.800			
	RMS error	0.376			

a. Lagx: the lagged months; HS: monthly hours of sunshine; Unit: temperature (degree centigrade), monthly hours of sunshine (10 hours).

b. The model including average wind velocity had a lower R-square (0.882) and higher RMS error (1.66) and it was not shown in the table. The purely autoregressive model had a R-square 0.788.

c. The model including rainfall had lower R-squares (0.874) and higher RMS errors (0.337) and it was not shown in the table. The purely autoregressive model had a R-square 0.714.

d. The model including rainfall or wind velocity had lower R-squares (0.859, 0.827) and higher RMS errors (0.334, 0.339) and they were not shown in the table. The purely autoregressive model had a R-square 0.753.

e. The model including rainfall had lower R-squares (0.892) and higher RMS errors (0.382) and it was not shown in the table. The purely autoregressive model had a R-square 0.818.

## Discussion

Our study provides a complete overview of the epidemiological features of re-emerging human brucellosis in mainland China from 2004 to 2010. The epidemics presented significant spatial heterogeneity in different regions of mainland China and areas experiencing severe epidemics were focused in northern, north-eastern and north-western China. Our analyses of factors influencing the pattern of spatial heterogeneity indicated that animal husbandry (mainly sheep and goats) as well as relevant geographic landscape (moderate elevation and a greater proportion of grassland) contributed significantly to the spatial pattern of the epidemics. Lower temperature and less sunshine in winter and spring, with time lags between 1–7 months occurring before the epidemic peaks in May, facilitated the local epidemics of human brucellosis.

Since the beginning of the 21st century, human brucellosis has been re-emerging in China and the number of human brucellosis cases reached a historic high in 2009. Males in the 30–50 year age group had the highest incidence: this is probably a reflection of the exposure through occupation of males to livestock in a pastoral economy where females are less exposed to livestock in their domestic duties [30,31]. In northern, north eastern and western China cases were found mainly among peasants and herdsman. In southern China and in urban areas, cases were found among other occupations such as urban workers, food service workers, and retired workers, where human brucellosis transmission could be associated with eating habits, or travel to or from endemic areas [23]. Distribution of human brucellosis in other studies confirms that cases are frequently clustered in occupational and food-related settings [32].

Using the multivariate Poisson regression analysis to understand the environmental and socioeconomic factors associated with the spatial-clustered distribution of human brucellosis, we found that higher incidence was associated with higher density of sheep and goats (rather than swine and cattle), as well as with an environmental landscape of moderately elevated grassland. Our results indicated that sheep and goats probably were the main animal hosts transmitting the diseases to humans in northern, north-eastern and western China, where the severest epidemics occurred [33]. Although sporadic outbreaks of cattle brucellosis were found during the surveillance period, it did not appear to be associated with a higher incidence of human brucellosis. This finding indicates that control measures targeted in those areas where there is exposure to sheep or goats would be particularly effective, even though swine and cattle also have the potential to transmit the disease to humans [34,35]. Analysis of the geographic landscape showed that in general, grassland and moderate elevation are also risk factors for human brucellosis, in an environment that is suitable habitat for farming sheep and goats. The exceptions to this were Qinghai, Tibet and western Sichuan in the Qinghai-Tibet plateau which have large areas of grassland of moderate elevation but a very low incidence of human brucellosis (0.78/100,000). However, the livestock here are predominantly yak and antelope, rather than sheep or goats. These animals are generally farmed by free-range feeding methods, thus minimising human-animal contact [36,37]. Based on the ADL modelling in the four provinces with the severest epidemics of human brucellosis, we showed that the temporal trend of monthly incidence was influenced by temperature and sunshine. Lower temperatures and less sunshine with 1–7 month time lags (around winter and spring) before the epidemic peak in May, were clearly associated with local epidemics. In zoonoses, changes in climatic factors naturally influence infection rates and population dynamics of animal hosts, as well as exposure of humans to infected animals [38]. Breeding of sheep and goats occurs in winter and early spring, increasing contact between animal stocks themselves at these times. Low temperature and less sunshine in winter and spring could prolong the time of indoor breeding for livestock, and increase the possibility of contact between animals and humans as well as between the groups of animals [39]. In addition, lambs and kids are born in winter or early spring, and human contact with amniotic fluid or infected young animals is a risk factor for human infection. This, combined with the 1–2 month incubation period for human brucellosis, and possible delayed diagnosis in the rural setting probably accounts for the peaks of human brucellosis in spring and summer [40-42].

In this study, we characterised the epidemiological features of human brucellosis and identified the environmental and socioeconomic factors associated with the spatial variations and the temporal trends of the disease. However, we recognise that there are certain limitations to the study. First, passive surveillance data are not as good as data collected from active surveillance. Some cases may go unreported because of their milder clinical symptoms, or some could be delayed in reporting because of delayed diagnosis in rural settings. In addition, tourism and general population movement could be complicating the reported pattern of transmission of human brucellosis. However, with the data available we have provided a comprehensive overview of the epidemiological features of human brucellosis in mainland China, and our findings provide hints as to where future intervention could be most effective.

## Conclusions

Our results indicate that attention should be focused on sheep and goat farming economies in areas of grassland with moderate elevation, especially in years when winter and spring are colder or have less sunshine than usual. Furthermore, the methodology we have employed may be helpful as a means of providing valuable information for risk evaluation of human brucellosis epidemics in the future.

## Abbreviations

ADL: Autoregressive distributed lag; AIC: Akaike’s information criterion; CCDC: Chinese center for disease control and prevention; CI: Confidence interval; HS: Hours of sunshine; NNDSS: National notifiable disease surveillance system; RH: Relative humidity; RMSE: Root mean square error; WV: Average wind velocity.

## Competing interests

All authors declare that they have no actual or potential competing financial interest.

## Authors’ contributions

WCC and LQF designed the study; YJL collected data; YJL and LQF did the statistical analyses and outcome assessment, and wrote the paper. XL and SL did the statistical analyses and the outcome assessment. All authors read and approved the final manuscript.

## Pre-publication history

The pre-publication history for this paper can be accessed here:

http://www.biomedcentral.com/1471-2334/13/547/prepub
